# Hypotension Requiring Vasopressor Support After Endovascular Thrombectomy: Predictors and Neurological Consequences

**DOI:** 10.3390/jcm15176521

**Published:** 2026-08-23

**Authors:** Justyna Zielińska-Turek, Dariusz Kosior, Jolanta Kołakowska, Małgorzata Dorobek

**Affiliations:** 1 Department of Neurology, National Medical Institute of the Ministry of the Interior and Administration, 137 Wołoska Str., 02-507 Warsaw, Poland; jzturek@gmail.com (J.Z.-T.);; 2 Mossakowski Medical Research Institute, Polish Academy of Sciences, 02-106 Warsaw, Poland; 3 Department of Sports Medicine, Centre of Postgraduate Medical Education, 00-416 Warsaw, Poland; 4 Department of Internal Medicine and Geriatric Cardiology, Centre of Postgraduate Medical Education, 00-416 Warsaw, Poland

**Keywords:** acute ischaemic stroke, endovascular thrombectomy, hypotension, blood pressure, anaesthesia, outcomes

## Abstract

**Background/Objectives**: Endovascular thrombectomy (EVT) has transformed the treatment of acute ischaemic stroke due to large-vessel occlusion, yet peri-procedural haemodynamic instability may compromise penumbral perfusion and neurological recovery. We assessed the frequency, determinants and clinical consequences of post-procedural hypotension after EVT. **Methods**: We retrospectively reviewed 201 consecutive adults who underwent endovascular thrombectomy for anterior-circulation large-vessel occlusion at a single tertiary centre over a period of eight years, from 1 January 2017 to 31 January 2025. Post-procedural hypotension was defined as hypotension requiring initiation of a continuous noradrenaline infusion within 24 h of the procedure. Comorbidities, anaesthetic modality (general anaesthesia [GA] or conscious sedation [CS]), National Institutes of Health Stroke Scale (NIHSS) and modified Rankin Scale (mRS) scores, and in-hospital mortality were recorded. Logistic regression identified independent predictors of hypotension. **Results**: Fifty-five patients (27.4%) developed post-procedural hypotension. They presented with more severe strokes (NIHSS 16.0 ± 5.0 vs. 13.7 ± 5.0; *p* = 0.002), had higher NIHSS scores at day 2 (14.0 ± 6.9 vs. 9.4 ± 6.7; *p* < 0.001) and day 7 (*p* = 0.003), and displayed markedly higher in-hospital mortality (50.9% vs. 20.5%; *p* < 0.001). In an ordinal analysis of the day 7 modified Rankin Scale with death coded as 6, hypotension was associated with a shift towards greater disability (common OR 2.57, 95% CI 1.40–4.72; *p* = 0.002). In an exploratory model, each additional 10 min of door-to-groin time was independently associated with hypotension (adjusted OR 1.10, 95% CI 1.03–1.18; *p* = 0.003). Independent predictors of hypotension were baseline NIHSS (adjusted OR 1.11 per point, 95% CI 1.04–1.19; *p* = 0.003) and active malignancy (adjusted OR 2.90, 95% CI 1.04–8.09; *p* = 0.042). Hypotension occurred with similar frequency under GA and CS (28.6% vs. 24.6%; adjusted OR 1.12, 95% CI 0.54–2.34; *p* = 0.766). **Conclusions**: Post-EVT hypotension is common and associated with poorer early neurological recovery and a more than two-fold higher in-hospital mortality rate. Its independent predictors were baseline stroke severity and active malignancy. Patients with severe stroke or active cancer may warrant intensified haemodynamic surveillance after thrombectomy. Procedural delay emerged as the only modifiable predictor identified and warrants prospective evaluation.

## 1. Introduction

Acute ischaemic stroke due to large-vessel occlusion (LVO) remains one of the leading causes of disability and mortality worldwide. Endovascular thrombectomy (EVT) has emerged as the standard-of-care for eligible patients because rapid mechanical removal of the occluding thrombus substantially improves functional outcomes compared with medical therapy alone [1]. After reperfusion, however, maintaining adequate cerebral perfusion is vital for salvaging the ischaemic penumbra. Experimental and clinical data suggest that the relationship between blood pressure (BP) and stroke outcome is U-shaped: both hypertension and hypotension have been associated with worse neurological outcomes. Reductions in systemic BP before recanalisation may compromise collateral circulation and exacerbate cerebral hypoperfusion at a time when cerebral autoregulation is impaired [2]. Conversely, severe hypertension can promote oedema, reperfusion injury and haemorrhagic conversion.

Current guidelines therefore encourage permissive hypertension before EVT and explicitly recommend correcting hypotension and hypovolaemia [3]. In practice, the American Heart Association/American Stroke Association suggests that systolic BP (SBP) may be tolerated up to ≤185/110 mm Hg before EVT (or <180/105 mm Hg in patients receiving intravenous thrombolysis) [3]. Once recanalisation has been achieved, optimal BP targets remain uncertain. Observational studies indicate that both high mean BP trajectories (>140 mm Hg) and marked BP variability after EVT are associated with increased mortality and symptomatic intracerebral haemorrhage [4]. Peri-procedural blood-pressure changes during mechanical thrombectomy have been linked to serious early complications, including symptomatic intracerebral haemorrhage and malignant cerebral oedema [5]. By contrast, a recent randomised trial comparing intensive (<120 mm Hg) vs. standard (140–180 mm Hg) post-EVT BP management was halted early because the intensive strategy increased early neurological deterioration and mortality [6]. Consequently, most experts currently recommend maintaining SBP within 140–180 mm Hg before recanalisation and 120–140 mm Hg after successful reperfusion, while avoiding aggressive BP lowering that might precipitate hypotension [7].

The choice of anaesthetic technique further complicates peri-procedural haemodynamic management. Conscious sedation (CS) and local anaesthesia are often easier and faster to administer, minimising delays to groin puncture; general anaesthesia (GA) provides airway protection and patient immobility but can introduce haemodynamic instability and procedural delays [8]. GA may impair mean arterial pressure during induction, potentially compromising collateral flow and the viability of the ischaemic penumbra [9]. Early observational registries suggested that GA was associated with increased mortality and worse functional outcomes compared with sedation, partly because GA patients tended to have more severe strokes and episodes of hypotension [10]. More recent randomised trials (SIESTA, GOLIATH and AnStroke) used protocol-driven BP targets and demonstrated no significant difference in functional outcomes between GA and sedation [11,12,13]. This is consistent with a recent Cochrane systematic review, which concluded that there is insufficient evidence to support one anaesthetic strategy over another owing to the limited number and quality of randomised trials [14]. These data imply that meticulous haemodynamic control, rather than the anaesthetic technique itself, determines outcome. Nonetheless, intraprocedural BP fluctuations and episodes of hypotension remain common with both GA and sedation [12]. Many centres target SBP 140–160 mm Hg (or 120–180 mm Hg) during EVT and use vasopressor infusions to maintain BP in the GA group [15].

Despite growing awareness of the potential harm of peri-procedural hypotension, there is no consensus on the optimal definition of hypotension in the EVT setting. Studies use disparate thresholds, ranging from absolute SBP cut-offs (e.g., <100 mm Hg) to relative declines (>10–30%) from baseline values. This variability reflects the broader perioperative literature, in which the Perioperative Quality Initiative (POQI) consensus recommends defining intraoperative hypotension as a mean arterial pressure below 60–70 mm Hg or a systolic pressure <100 mm Hg, and acknowledges that relative reductions of ≥20% from baseline are clinically meaningful [16]. Illustrating this problem, an analysis that applied multiple published definitions to a single surgical cohort found that the incidence of intraoperative hypotension varied more than tenfold depending solely on the definition applied [17]. Moreover, the concept of a “normal” intra-operative blood pressure remains ill-defined; moderate deviations from baseline may not uniformly predict adverse outcomes in general surgical populations [18]. The lack of standardisation hampers comparisons between studies and obscures which thresholds are most relevant for patients undergoing EVT.

Given these uncertainties, we sought to quantify the frequency and determinants of post-procedural hypotension in a contemporary cohort of EVT-treated patients and to evaluate its association with early neurological recovery and in-hospital mortality. By elucidating the predictors and clinical consequences of hypotension, our study aims to inform haemodynamic management strategies and contribute to ongoing efforts to individualise blood-pressure targets after reperfusion.

## 2. Materials and Methods

### 2.1. Study Design and Patient Selection

This was a retrospective, single-centre observational cohort study conducted at the Department of Neurology, National Medical Institute of the Ministry of the Interior and Administration, Warsaw, Poland, a tertiary comprehensive stroke centre. The study population comprised a single consecutive cohort; patients were not allocated to treatment groups. Post-procedural hypotension was an observed exposure, and the comparison groups reported throughout represent subgroups of this cohort defined by whether the prespecified criterion was met within 24 h of the procedure.

We screened all consecutive patients who underwent endovascular thrombectomy for acute ischaemic stroke between 1 January 2017 and 31 January 2025 (*n* = 260).

Patients were eligible if they met all of the following criteria: (1) age ≥ 18 years; (2) acute ischaemic stroke attributable to angiographically confirmed large-vessel occlusion; (3) treatment with mechanical thrombectomy, with or without preceding intravenous thrombolysis; and (4) availability of peri-procedural haemodynamic records covering the first 24 h after the procedure.

**Figure 1:**
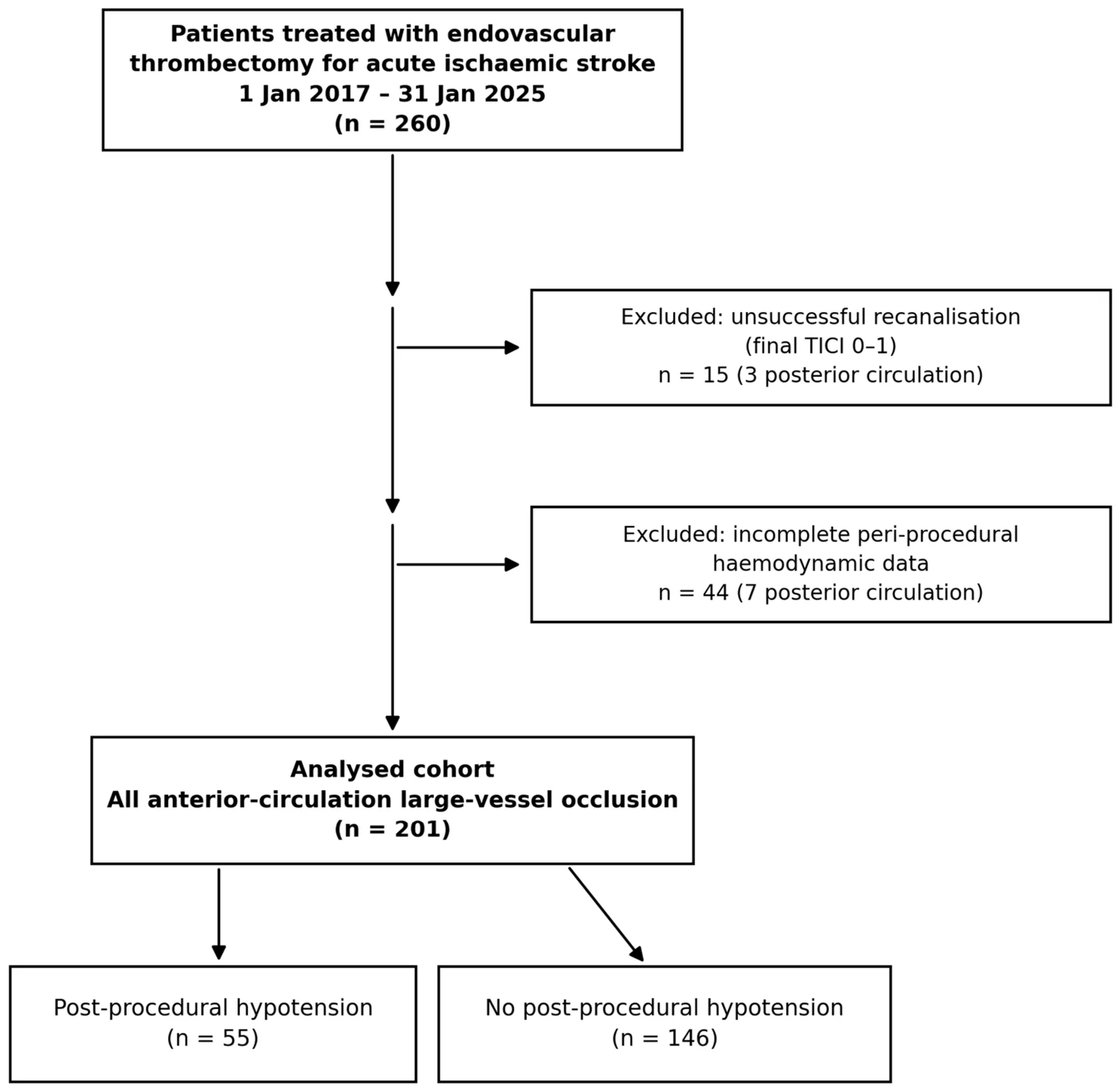
Flow of patients through the study. TICI = Thrombolysis in Cerebral Infarction.

Patients were excluded if they had (1) unsuccessful recanalisation, defined as a final Thrombolysis in Cerebral Infarction (TICI) grade of 0 or 1 (*n* = 15), or (2) incomplete peri-procedural haemodynamic records, precluding classification of hypotension status (*n* = 44). The final analysable cohort comprised 201 patients (Figure 1).

Occlusion territory was not itself an exclusion criterion. Ten patients with posterior-circulation occlusion were identified during screening, and all ten met one of the exclusion criteria above (three with TICI 0–1 and seven with incomplete haemodynamic records). The analysed cohort therefore consists exclusively of patients with anterior-circulation large-vessel occlusion.

### 2.2. Endovascular Procedure

All 201 patients underwent mechanical thrombectomy for anterior-circulation large-vessel occlusion. Procedures were performed in a dedicated neuroangiography suite by one of five experienced interventional neuroradiologists practising at the centre, each procedure being carried out by a single operator working with the institutional neuroanaesthesia team. Access was transfemoral, with a long guide catheter (NeuronMAX, Penumbra Inc., Alameda, CA, USA) positioned in the parent vessel.

Revascularisation was performed using a combined stent retriever and aspiration technique as the first-line approach: a microcatheter was navigated across the thrombus, a stent retriever (Trevo, Stryker Neurovascular, Fremont, CA, USA) deployed within it and an aspiration catheter (Sofia 6 Plus, MicroVention Inc., Aliso Viejo, CA, USA) advanced to the proximal face of the clot before retrieval. On subsequent passes, aspiration alone was used at the operator’s discretion. The number of passes was determined intraprocedurally and documented descriptively in the angiographic reports, but was not extracted in a structured form and could not be analysed. The femoral puncture site was closed with a vascular closure device (Perclose ProStyle, Abbott Vascular, Santa Clara, CA, USA) and compressive dressing.

Technique and device platform were standardised institutionally across operators, but operator identity was not recorded in the analytical dataset, so operator-level variation in procedural conduct could not be examined; this is addressed in Section Limitations.

Intravenous thrombolysis was administered before thrombectomy as a bridging therapy in patients presenting within the therapeutic window and without contraindication; 113 patients (56.2%) received bridging thrombolysis. Thrombolysis was recorded as a co-intervention and was not the treatment under evaluation.

Angiographic outcome was graded on the modified Thrombolysis in Cerebral Infarction scale on the final angiographic run by the treating interventionalist and recorded in the procedure report. During preparation of this revision, all angiographic grades were re-verified against the source radiology reports; fifteen previously unrecorded grades were retrieved and four erroneous entries were corrected. Where a report gave two adjacent grades, the lower grade was assigned (2c/3 → 2c; 2b/c → 2b; 2b/2a → 2a); a sensitivity analysis assigning the higher grade is reported in the Results.

Procedure duration, induction agents and the number of thrombectomy passes were not recorded consistently across the study period and could not be analysed.

### 2.3. Data Collection and Definitions

Treatment-related variables comprised administration of intravenous alteplase and the choice of anaesthetic modality (GA vs. CS), which was retrieved for every patient from the original anaesthetic records. Procedure duration, induction agents and the number of thrombectomy passes were not recorded consistently across the study period and were therefore not analysed.

Blood pressure measurement was standardised across the study period. Prior to induction, three consecutive systolic BP readings were obtained using either a calibrated non-invasive cuff or a radial arterial line; their average served as the pre-procedural baseline. During EVT, patients underwent continuous haemodynamic monitoring via an invasive arterial catheter whenever feasible; if arterial access was unavailable, automated non-invasive readings were recorded at one-minute intervals. Monitoring continued for at least 24 h after the procedure in a dedicated stroke unit. When available, we documented the duration and timing of hypotensive episodes, the cumulative dose of vasopressors and the use of volume resuscitation, although these variables were not included in the primary analysis because of incomplete data.

### 2.4. Anaesthesia Management

All procedures were performed in collaboration with neuroanaesthesiologists. The decision to employ GA vs. CS was made case by case, considering the patient’s level of consciousness, ability to cooperate, aspiration risk and anticipated procedural complexity. For CS, local anaesthesia at the femoral puncture site was combined with titrated intravenous sedation (typically midazolam and/or dexmedetomidine), allowing spontaneous ventilation and neurological examination. GA was induced with intravenous propofol or etomidate combined with a short-acting opioid, followed by neuromuscular blockade and endotracheal intubation and maintained with volatile agents (sevoflurane or desflurane) or continuous propofol infusion according to institutional protocol. In both modalities, anaesthesiologists aimed to keep SBP between 140 and 180 mm Hg during the procedure and mean arterial pressure ≥70 mm Hg using fluid boluses, vasopressors and, when necessary, inotropic support. If CS proved inadequate because of agitation or airway compromise, conversion to GA was performed emergently; these cases were analysed within the GA group.

### 2.5. Definition of the Exposure

The exposure of interest was post-procedural hypotension requiring vasopressor support, defined a priori as the initiation of a continuous intravenous noradrenaline infusion for hypotension at any point during the first 24 h after thrombectomy. Patients were classified as exposed if such an infusion was recorded in the anaesthetic or intensive-care chart, and as unexposed if no vasopressor infusion was administered during that period.

This definition captures a clinically actionable event—haemodynamic compromise judged by the treating team to require pharmacological correction—rather than a numerical threshold. It differs from definitions based on absolute or relative systolic pressure cut-offs used in the perioperative literature [16], and its limitations are addressed in Section Limitations.

To characterise the depth of the hypotensive episodes, the lowest recorded systolic and diastolic pressures during the first 24 h were retrieved for patients who received vasopressor support (available for 54 of 55). These values are reported descriptively in the Results and were not used to define the exposure. Corresponding measurements were not retrievable for patients who did not receive vasopressor support, and pre-procedural baseline pressures were not available in a form permitting calculation of relative decline; neither an absolute nor a relative threshold definition could therefore be applied to the cohort. The duration of hypotensive episodes, cumulative vasopressor dose and the use of volume resuscitation were likewise not captured and could not be analysed.

### 2.6. Clinical Variables and Outcome Measures

The National Institutes of Health Stroke Scale (NIHSS) was scored on admission, immediately prior to the endovascular procedure, by the treating neurologist. This admission score is referred to throughout as the baseline (day 1) NIHSS and serves both as a covariate and as the first point of the NIHSS trajectory.

Recorded variables comprised age, sex, hypertension, diabetes mellitus, atrial fibrillation, current smoking, chronic kidney disease and active malignancy, together with anaesthetic modality, administration of bridging thrombolysis and final TICI grade.

Two procedural time metrics were retrieved from institutional records: door-to-needle time (DNT), from hospital arrival to administration of intravenous alteplase, recorded for all 113 patients who received bridging thrombolysis; and door-to-groin time (DGT), from hospital arrival to arterial puncture, recorded for 179 patients. One DGT entry of 0 min was implausible and was treated as missing, leaving 22 patients without a DGT value.

The primary outcome was the occurrence of post-procedural hypotension requiring vasopressor support. Secondary endpoints comprised (1) early neurological recovery, defined as the change in NIHSS score from baseline to day 2 and day 7; (2) functional independence at day 7, defined as mRS 0–2; (3) disability measured by the mRS and the Barthel Index at day 7; and (4) in-hospital mortality. Patients who died before day 7 had no day 7 assessment; those who survived to day 7 and died later were assessed at day 7 and their recorded scores retained. Data were extracted from institutional records by an investigator not involved in peri-procedural care. Because clinical NIHSS and mRS assessments were performed prospectively during hospitalisation by the treating team, formal blinding to anaesthetic modality and blood-pressure course was not possible. Baseline ASPECTS was documented for only 35 patients (17.4%) and was therefore not analysed.

### 2.7. Statistical Analysis

Continuous variables were assessed for normality using the Shapiro–Wilk test and are presented as the mean ± standard deviation together with the median and interquartile range; categorical variables are presented as counts with the denominator stated explicitly and the corresponding percentage. Between-group comparisons used the Mann–Whitney U test for continuous variables and Fisher’s exact test for categorical variables; Fisher’s exact test was preferred over the χ^2^ test throughout because several cells contained fewer than ten observations.

Variables associated with post-procedural hypotension at *p* < 0.10 in univariable analyses and clinically relevant covariates (age, sex) were entered into a multivariable logistic regression model; anaesthetic modality was included a priori given its clinical importance. Adjusted odds ratios (aORs) with 95% confidence intervals quantified the strength of associations. The model included six covariates for 55 events, giving 9.2 events per variable.

Primary analyses of day 7 outcomes were complete-case, and the number of patients contributing to each comparison is reported alongside the corresponding estimate. Because these analyses are necessarily conditioned on survival to assessment, two prespecified sensitivity analyses were performed. First, all day 7 outcomes were repeated, with death before assessment assigned the worst possible score (mRS 6, Barthel Index 0). Second, the distribution of the day 7 mRS across the cohort was analysed by proportional-odds ordinal logistic regression (shift analysis) adjusted for age, sex and admission NIHSS, with death coded as mRS 6; a common odds ratio above 1 indicates a shift in the entire distribution towards greater disability.

Successful reperfusion was achieved in 198 of 201 patients (98.5%). Because unsuccessful reperfusion occurred in only three patients, this variable was near-constant and could not be entered into the multivariable model; the reasons are set out in the Results.

A two-sided *p* < 0.05 was considered statistically significant. All analyses were conducted with SPSS version 28.0 (IBM Corp., Armonk, NY, USA) and verified independently.

## 3. Results

### 3.1. Baseline Characteristics

**Figure 2:**
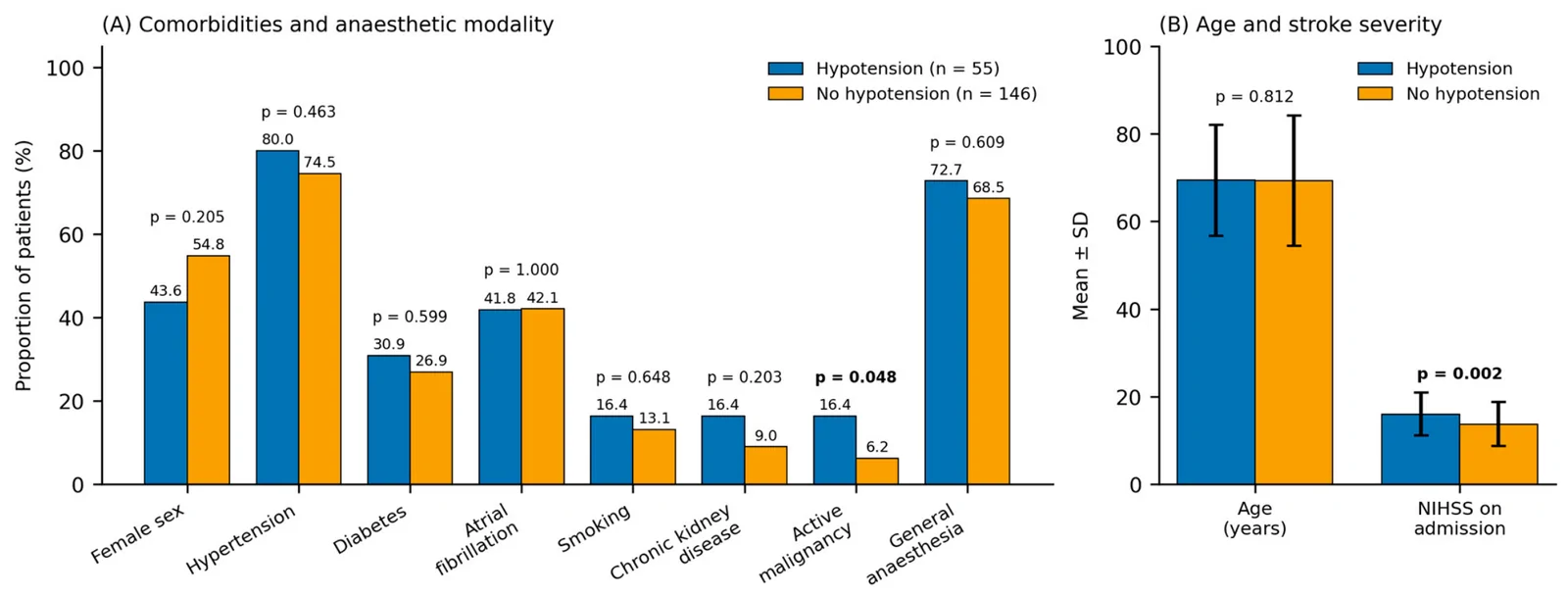
Baseline characteristics by post-procedural hypotension status. (**A**) Comorbidities and anaesthetic modality; (**B**) age and stroke severity (age and NIHSS on admission, mean ± SD).

Among 201 included patients, the mean age was 69.3 ± 14.3 years and 104 (51.7%) were women. Baseline characteristics stratified by hypotension status are summarised in Table 1. Patients who developed hypotension (n = 55) presented with more severe strokes (median NIHSS 17 [IQR 13–20] vs. 14 [11,12,13,14,15,16,17,18]; *p* = 0.002) and more frequently had an active malignancy (16.4% vs. 6.2%; *p* = 0.048). Age, sex, anaesthetic modality and the prevalence of hypertension, diabetes, atrial fibrillation, smoking and chronic kidney disease did not differ significantly between groups (Figure 2). The median door-to-needle time among the 113 patients who received bridging thrombolysis was 55 min (IQR 39–83; range 4–190), and the median door-to-groin time among the 179 patients with a valid record was 143 min (IQR 116–176; range 26–356). Both were longer in patients who subsequently developed post-procedural hypotension: DNT 76 min (IQR 44–98) vs. 54 min (IQR 36–75), *p* = 0.021; DGT 158 min (IQR 125–196) vs. 140 min (IQR 113–167), *p* = 0.021. The frequency of hypotension was highest in the longest quartile of door-to-groin time, rising from 20.0% (9/45) in the shortest quartile to 41.9% (18/43) in the longest, although the gradient was not strictly monotonic. Successful reperfusion (TICI 2b–3) was achieved in 198 of 201 patients (98.5%).

### 3.2. Peri-Procedural Variables and Outcomes

**Figure 3:**
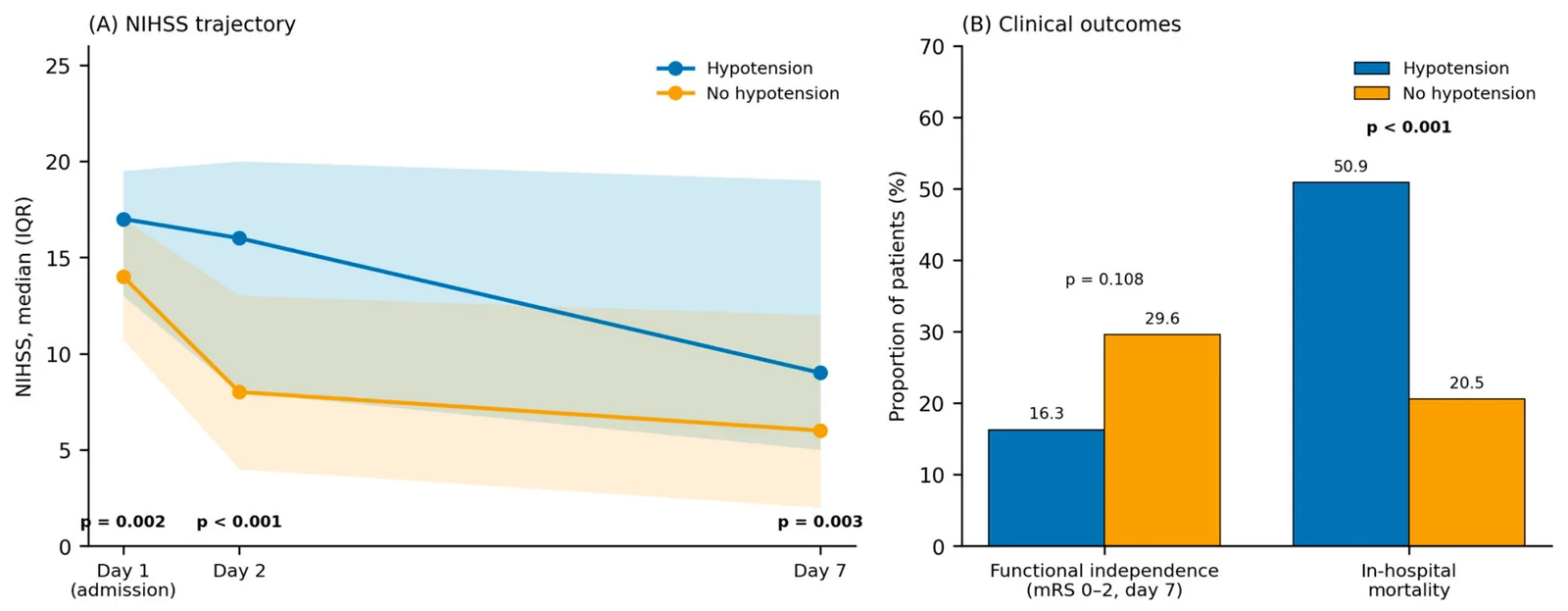
Neurological course and outcomes by post-procedural hypotension status. (**A**) NIHSS trajectory from admission (day 1) to day 7, shown as median (IQR); (**B**) clinical outcomes: functional independence (mRS 0–2) at day 7 and in-hospital mortality.

Table 2 compares the neurological course and in-hospital outcomes of patients with and without hypotension. Patients experiencing hypotension had significantly higher NIHSS scores at every time point: day 1 (16.04 ± 4.95 vs. 13.74 ± 4.99; *p* = 0.002), day 2 (14.02 ± 6.85 vs. 9.42 ± 6.66; *p* < 0.001) and day 7 (12.26 ± 9.06 vs. 8.37 ± 9.04; *p* = 0.003). Disability at day 7 was correspondingly greater (mRS 3.77 ± 1.44 vs. 3.11 ± 1.71; *p* = 0.023). Functional independence (mRS 0–2) was reached by 16.3% of hypotensive patients vs. 29.6% of normotensive patients, a difference that did not attain statistical significance (*p* = 0.108), and the Barthel Index at day 7 showed a similar non-significant trend (37.6 ± 37.6 vs. 49.0 ± 39.5; *p* = 0.087). In-hospital mortality was markedly higher among patients with hypotension (28/55, 50.9% vs. 30/146, 20.5%; *p* < 0.001) (Figure 3). Among the 55 patients who received vasopressor support, the median lowest recorded systolic pressure during the first 24 h (available for 54) was 110 mm Hg (IQR 96–117; range 60–120) and the median lowest diastolic pressure was 60 mm Hg (IQR 55–70; range 30–90). Sixteen of these fifty-four patients (29.6%) had a nadir systolic pressure below 100 mm Hg and seven (13.0%) had one below 90 mm Hg, while in thirty (55.6%), the nadir remained at or above 110 mm Hg.

Because day 7 assessments were unavailable for patients who died before that point, two prespecified sensitivity analyses were performed (Table 3). Day 7 assessments were missing for 12 of 55 patients with hypotension (10 died before assessment, 2 had incomplete records) and for 21 of 146 patients without hypotension (7 died before assessment, 14 had incomplete records).

When death before assessment was assigned the worst possible score, functional independence was reached by 7 of 53 patients with hypotension (13.2%) vs. 37 of 132 without hypotension (28.0%; *p* = 0.036), the day 7 mRS was 4.19 ± 1.57 vs. 3.27 ± 1.79 (*p* = 0.001) and the Barthel Index was 30.5 ± 36.9 vs. 46.2 ± 40.0 (*p* = 0.010). Both the functional-independence and Barthel comparisons, which were non-significant in the complete-case analysis, reached significance once survivorship was accounted for; the complete-case estimates are therefore conservative.

**Figure 4:**
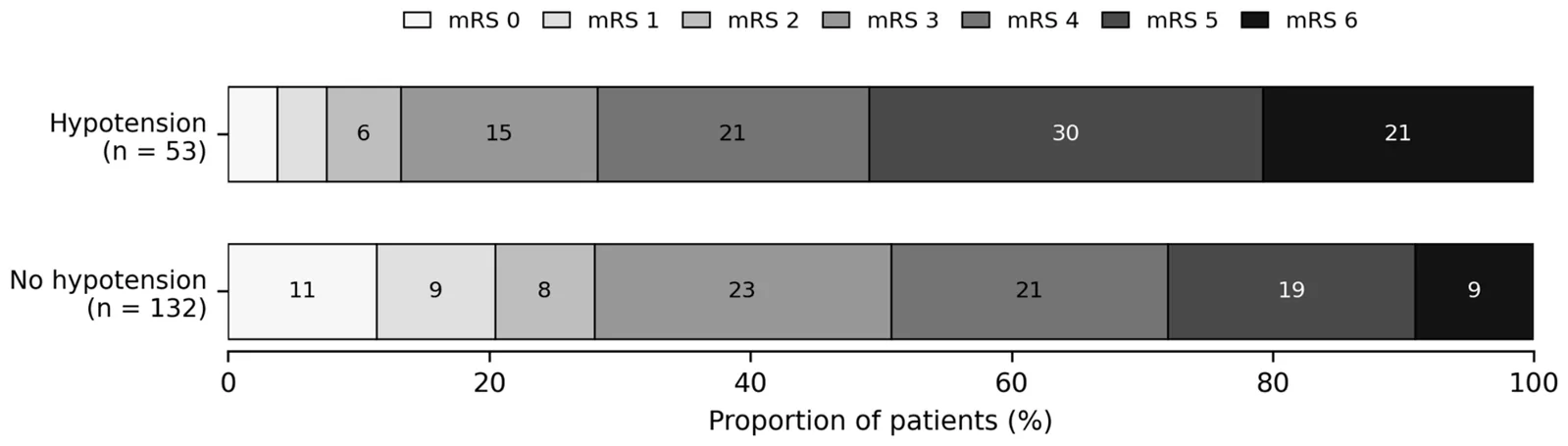
Distribution of the modified Rankin Scale at day 7 by post-procedural hypotension status, with death before assessment coded as mRS 6. Shading from light to dark denotes increasing mRS score (0–6), as indicated in the legend; the numbers within the bars are percentages of the group.

In the ordinal shift analysis of the full day 7 mRS distribution with death coded as mRS 6, post-procedural hypotension was associated with a shift in the entire distribution towards greater disability (common OR 2.57, 95% CI 1.40–4.72; *p* = 0.002) after adjustment for age, sex and NIHSS on admission (n = 184). The distribution is shown in Figure 4.

Anaesthetic modality was documented for all 201 patients: 140 (69.7%) underwent EVT under GA and 61 (30.3%) under CS. Hypotension occurred in 40 GA patients (28.6%) and 15 CS patients (24.6%), a difference that was not statistically significant (*p* = 0.609, Fisher’s exact test). In-hospital mortality was nevertheless higher in the GA group (35.7% vs. 13.1%; *p* = 0.001), consistent with the preferential use of GA in older and more severely affected patients.

In univariable analysis (Table 4), baseline NIHSS (OR 1.10 per point, 95% CI 1.03–1.17; *p* = 0.005) and active malignancy (OR 2.96, 95% CI 1.11–7.90; *p* = 0.031) were associated with the occurrence of hypotension, while chronic kidney disease showed a non-significant trend (OR 1.99, 95% CI 0.80–4.95; *p* = 0.141). Age, sex, hypertension, diabetes, atrial fibrillation and smoking were not associated with hypotension, nor was general anaesthesia (OR 1.23, 95% CI 0.62–2.44; *p* = 0.561).

### 3.3. Anaesthesia Modality

**Figure 5:**
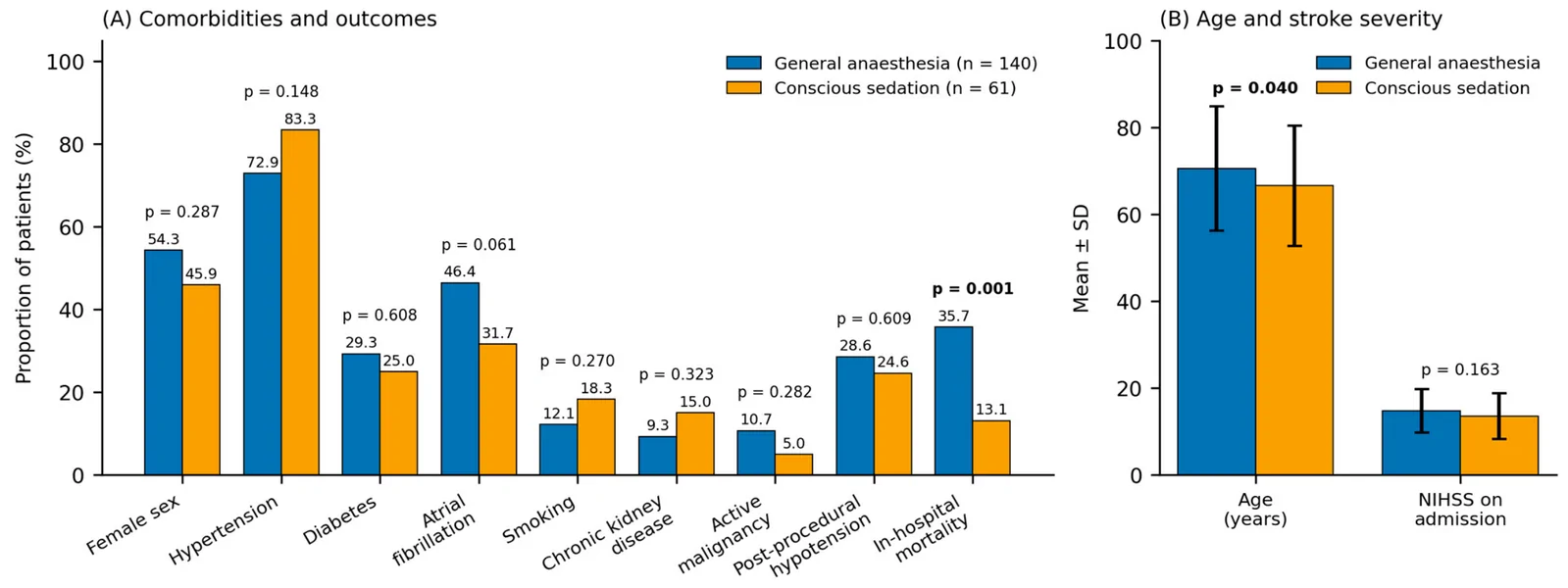
Patient and stroke characteristics by anaesthetic modality. (**A**) Comorbidities and outcomes; (**B**) age and stroke severity (age and NIHSS on admission, mean ± SD).

Baseline characteristics according to anaesthetic modality are shown in Table 5. Patients receiving GA were significantly older (70.52 ± 14.37 vs. 66.57 ± 13.84 years; *p* = 0.040) and showed a trend towards more frequent atrial fibrillation (46.4% vs. 31.7%; *p* = 0.061) and more severe strokes (NIHSS 14.74 ± 4.94 vs. 13.52 ± 5.31; *p* = 0.163), consistent with the preferential selection of GA for more complex patients. The prevalence of hypertension, diabetes, smoking and active malignancy was comparable between groups (Figure 5).

### 3.4. Predictors of Hypotension

**Figure 6:**
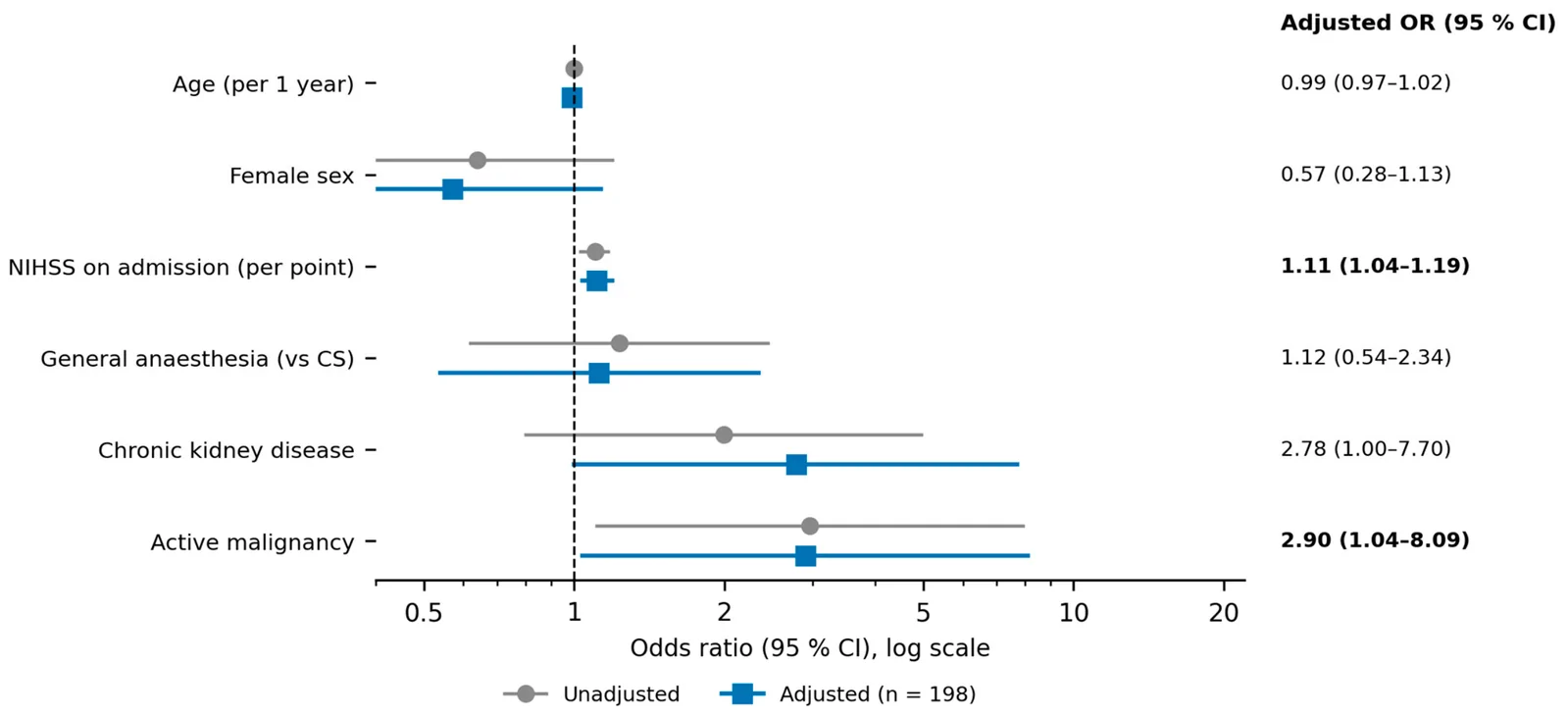
Unadjusted and adjusted predictors of post-procedural hypotension. Forest plot on a logarithmic scale; squares denote adjusted odds ratios from the multivariable model (n = 198), circles the corresponding unadjusted estimates. The dashed line marks an odds ratio of 1. Estimates set in bold are statistically significant (*p* < 0.05).

In the multivariable model (Table 6, n = 198), baseline NIHSS (aOR 1.11 per point, 95% CI 1.04–1.19; *p* = 0.003) and active malignancy (aOR 2.90, 95% CI 1.04–8.09; *p* = 0.042) were independent predictors of post-procedural hypotension, with chronic kidney disease at the margin of significance (aOR 2.78, 95% CI 1.00–7.70; *p* = 0.050). Neither age, sex nor anaesthetic modality was associated with the outcome; general anaesthesia showed no independent effect after adjustment (aOR 1.12 (0.54–2.34); *p* = 0.766) (Figure 6).

In univariable analysis, each additional 10 min of door-to-groin time was associated with the occurrence of post-procedural hypotension (OR 1.08, 95% CI 1.02–1.15; *p* = 0.014), as was each additional 10 min of door-to-needle time among thrombolysed patients (OR 1.17, 95% CI 1.04–1.32; *p* = 0.011).

### 3.5. Exploratory Analysis: Procedural Delay

**Figure 7:**
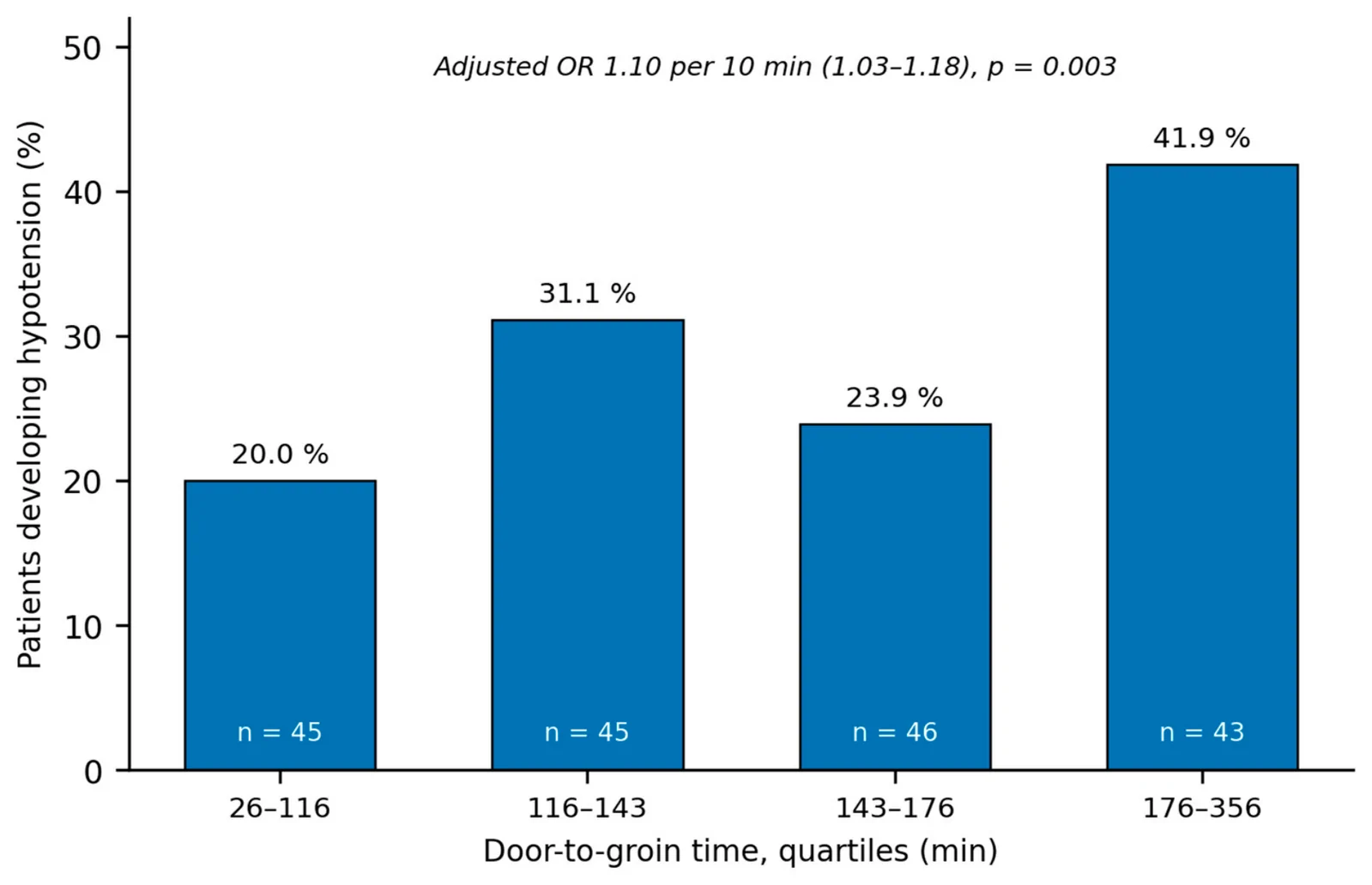
Frequency of post-procedural hypotension by quartile of door-to-groin time. Exploratory analysis; adjusted OR 1.10 per 10 min (95% CI 1.03–1.18), *p* = 0.003.

In an exploratory multivariable model restricted to the 177 patients with complete data (Table 7), door-to-groin time remained independently associated with hypotension (aOR 1.10 per 10 min, 95% CI 1.03–1.18; *p* = 0.003), as did NIHSS on admission (aOR 1.13, 95% CI 1.05–1.22; *p* = 0.002). In this subcohort, active malignancy (aOR 2.78, 95% CI 0.98–7.91; *p* = 0.056) and chronic kidney disease (aOR 2.84, 95% CI 0.95–8.50; *p* = 0.062) did not reach the significance threshold. Fitting the prespecified model to the same 177 patients without door-to-groin time gave an adjusted odds ratio of 2.69 (95% CI 0.96–7.52; *p* = 0.059) for active malignancy, indicating that the loss of significance reflects the smaller sample rather than the addition of the time covariate. The frequency of post-procedural hypotension across quartiles of door-to-groin time is shown in Figure 7.

## 4. Discussion

This study provides a contemporary assessment of haemodynamic disturbances following endovascular thrombectomy in a consecutive series of 201 patients. We observed that post-procedural hypotension occurred in more than one-quarter of cases and was not a benign event: patients who developed hypotension exhibited poorer early neurological recovery, greater disability at day 7 and a more than two-fold higher risk of in-hospital death compared with those who remained normotensive. In multivariable analysis, the independent predictors of hypotension were baseline stroke severity and active malignancy, whereas anaesthetic modality showed no association in either unadjusted or adjusted analyses. These findings shift the emphasis from procedural factors towards patient-level vulnerability as the principal determinant of peri-procedural haemodynamic instability.

Several features of these data call for a cautious, association-based interpretation rather than a causal one. Patients who became hypotensive presented with more severe strokes at baseline (median NIHSS 17 vs. 14), and baseline severity is itself a powerful determinant of both haemodynamic fragility and poor outcome. Hypotension may therefore represent, at least in part, a marker of more extensive cerebral injury and greater systemic illness rather than an independent cause of the adverse outcomes observed. Reverse causation is also plausible, in that evolving infarction, cerebral oedema and autonomic dysregulation can themselves precipitate blood-pressure instability. Although multivariable adjustment for stroke severity and other measured covariates attenuates this concern, it cannot eliminate residual and unmeasured confounding inherent to a retrospective design. Our findings should thus be read as hypothesis-generating: they identify hypotension as a robust prognostic marker and a biologically plausible therapeutic target, but they cannot establish that preventing hypotension will, by itself, improve outcomes.

The link between hypotension and adverse neurological outcomes has a solid physiological basis. Cerebral autoregulation is often impaired in the setting of large-vessel occlusion, rendering cerebral blood flow dependent on systemic perfusion pressures. Even transient reductions in systemic blood pressure can compromise collateral circulation and exacerbate cerebral ischaemia, whereas sustained hypertension may promote cerebral oedema and haemorrhagic conversion [2]. Consistent with these pathophysiological considerations, observational and mechanistic studies demonstrate that both absolute and relative decreases in blood pressure during thrombectomy are associated with larger infarct volumes and worse functional outcomes [4,5].

Our data also inform the ongoing debate surrounding anaesthetic strategy for EVT. Conscious sedation is attractive because it minimises delays to arterial puncture and permits intermittent neurological assessment, but it may be associated with patient movement and airway compromise. GA affords airway control and immobility but introduces the haemodynamic effects of induction agents and positive-pressure ventilation [8]. Early registry data suggested worse outcomes with GA, possibly due to more severe baseline strokes and hypotension [10]; randomised trials such as SIESTA, GOLIATH and AnStroke, employing strict blood-pressure protocols, subsequently showed no significant difference in functional outcomes between GA and CS [11,12,13], and a Cochrane review concluded that the available evidence does not support one anaesthetic strategy over another [14]. Our findings are consistent with that literature. Anaesthetic modality was documented for the entire cohort (140 GA, 61 CS), and hypotension occurred with similar frequency under both techniques (28.6% vs. 24.6%; OR 1.23, 95% CI 0.62–2.44; *p* = 0.561), with no independent effect after adjustment (aOR 1.12 (0.54–2.34); *p* = 0.766). In-hospital mortality was nevertheless higher in the GA group (35.7% vs. 13.1%; *p* = 0.001). This most plausibly reflects confounding by indication rather than an effect of anaesthesia itself, since patients selected for GA were significantly older and tended to have more severe strokes and more frequent atrial fibrillation—that is, GA marks a sicker population rather than causing the excess mortality. Taken together, these observations support the view that meticulous haemodynamic management, rather than the anaesthetic technique per se, is what matters for the patient.

Active malignancy emerged as an independent predictor of post-procedural hypotension (aOR 2.90, 95% CI 1.04–8.09), a finding that has not, to our knowledge, been reported previously in the EVT setting. Several mechanisms could plausibly contribute. Patients with cancer frequently exhibit reduced intravascular volume and impaired autonomic and cardiovascular reserve as a consequence of the disease itself, of cachexia, or of cardiotoxic chemotherapy and radiotherapy. Cancer-associated hypercoagulability and systemic inflammation may additionally alter vascular tone and endothelial function, and occult sepsis or adrenal insufficiency may further predispose to haemodynamic instability. Registry and meta-analytic data consistently show that patients with active malignancy achieve functional independence less often and die more frequently after thrombectomy than those without cancer, despite comparable rates of successful reperfusion [19,20]; haemodynamic instability may therefore represent one mechanism through which malignancy translates into poorer neurological recovery after EVT. This association was robust to adjustment for age, sex, stroke severity, renal function and anaesthetic modality, but rests on a modest number of events (18 patients with active malignancy) and requires confirmation in larger cohorts. Chronic kidney disease showed a comparable association at the margin of significance (aOR 2.78, 95% CI 1.00–7.70), and may reflect similar mechanisms of impaired volume regulation.

An additional challenge in interpreting the literature is the absence of a standardised definition of hypotension in the stroke setting. Perioperative medicine commonly employs absolute thresholds (e.g., SBP < 90–100 mm Hg or MAP < 60–70 mm Hg) and relative declines (>20–30% from baseline) [16], but stroke studies vary widely in the cut-offs used, hindering cross-study comparisons. We did not apply a numerical threshold; our exposure was the decision to initiate vasopressor support, which is why comparison with threshold-based studies must be made with caution. Whether lower or higher thresholds would better discriminate patients at risk of neurological deterioration remains uncertain. Future research should aim to harmonise definitions and explore the dose–response relationship between hypotension magnitude, duration and cerebral tissue injury using high-resolution blood-pressure recordings and advanced imaging markers.

Our data also illustrate why threshold-based definitions transfer poorly from the perioperative to the stroke setting. Among patients in whom the treating team judged haemodynamic support to be necessary, the nadir systolic pressure remained at or above 110 mm Hg in more than half, and fewer than a third fell below the 100 mm Hg threshold commonly applied in perioperative studies [16]. In a population in which cerebral autoregulation is impaired and perfusion of the penumbra is pressure-dependent, clinicians evidently intervened at pressures that would be considered unremarkable after elective surgery. Whether this reflects appropriate caution or an unnecessarily high treatment threshold cannot be determined from these data, but it suggests that pressures conventionally regarded as normal may not be adequate after reperfusion, and that definitions of hypotension for this population require empirical derivation rather than borrowing.

A key limitation of the present study is the absence of 90-day functional outcome data assessed by the modified Rankin Scale (mRS). The 90-day mRS is the accepted primary endpoint in stroke intervention trials and captures the full trajectory of neurological recovery, including late improvements that occur beyond the acute hospitalisation period [1]. Our analysis was confined to in-hospital outcomes—NIHSS at days 1, 2 and 7, mRS and Barthel Index at day 7, and in-hospital mortality—which, while clinically informative, may not fully reflect the ultimate functional burden attributable to post-EVT hypotension. Several lines of evidence suggest that early haemodynamic disturbances exert effects extending well beyond the acute phase. A large individual-patient-data meta-analysis demonstrated that higher post-EVT blood-pressure variability within the first 24 h was independently associated with 90-day disability and mortality, even after adjustment for mean blood-pressure levels [4]. Similarly, Wiącek et al. showed that the degree of intraprocedural blood-pressure drop correlated with final infarct volume, a strong determinant of 90-day functional outcome [21]. The association between early neurological status and 90-day outcome is well established, with early NIHSS and discharge mRS both acting as robust predictors of 90-day mRS in patients undergoing EVT [22]. Given that hypotensive patients in our cohort had substantially higher NIHSS scores at days 2 and 7 and greater disability at day 7 (mRS 3.77 ± 1.44 vs. 3.11 ± 1.71; *p* = 0.023), it is plausible that these early differences would also be reflected in long-term functional outcomes. Although the complete-case comparison of functional independence at day 7 did not reach significance, that analysis was conditioned on survival; when patients who died before assessment were assigned the worst outcome, the difference became significant (13.2% vs. 28.0%; *p* = 0.036), and the ordinal analysis of the full mRS distribution showed a clear shift towards disability (common OR 2.57; *p* = 0.002). Future studies should incorporate structured 90-day follow-up, ideally via standardised telephone or clinic-based mRS assessments, to quantify the long-term functional consequences of post-EVT hypotension and to determine whether prompt haemodynamic intervention can meaningfully improve 90-day outcomes.

From a clinical standpoint, our findings reinforce the importance of personalised haemodynamic management during and after EVT. Current guidelines advocate permissive hypertension prior to reperfusion, with SBP targets of 140–180 mm Hg, and a more conservative range of 120–140 mm Hg following successful recanalisation [7]. Our data do not permit recommendations about specific pressure thresholds, since the exposure we measured was the decision to initiate vasopressor support rather than a numerical value. They do, however, indicate which patients are most likely to require that support: those presenting with severe stroke, those with active malignancy, and those in whom the interval from arrival to arterial puncture is prolonged. The first two identify who should be watched; the last is the only predictor amenable to system-level intervention. These groups may warrant intensified haemodynamic surveillance, a lower threshold for invasive arterial monitoring and earlier vasopressor support, irrespective of the anaesthetic technique chosen [15]. Our findings sit within a substantial body of work linking peri-procedural blood pressure to outcome after thrombectomy, of which three studies bear most directly on the present analysis. Anadani and colleagues, studying patients after successful revascularisation, described a relationship in which both markedly elevated and markedly reduced post-procedural systolic pressures predicted worse functional outcome, consistent with the U-shaped relationship invoked above [23]. In an individual patient data meta-analysis, Katsanos and colleagues found that higher systolic pressure during the first 24 h after thrombectomy was associated with reduced odds of functional independence and with increased mortality, the association being most pronounced in patients in whom reperfusion had been achieved [24]. Mistry and colleagues, in a secondary analysis of the BEST study, showed that blood-pressure variability within the first 24 h predicted neurological outcome independently of mean pressure, indicating that the stability of perfusion pressure matters and not only its absolute level [25].

Our data complement rather than replicate these observations. Because our exposure was the clinical decision to initiate vasopressor support rather than a numerical threshold, it identifies the point at which treating teams judged cerebral perfusion to be inadequate, and that point lay, in most cases, well above the pressures at which these studies located risk.

### Limitations

Several limitations warrant consideration. First, the retrospective design precludes causal inference. Because the exposure was not randomly assigned, our findings are vulnerable to reverse causation—evolving infarction, cerebral oedema and autonomic dysregulation may precipitate haemodynamic instability rather than result from it—and to residual confounding. This applies with particular force to the association with in-hospital mortality: vasopressor support is administered preferentially to the most severely affected patients, and multivariable adjustment attenuates but cannot eliminate confounding by indication.

Second, and most importantly, the exposure was hypotension requiring vasopressor support rather than a measured blood-pressure threshold. Numerical blood-pressure values were not retrievable in a time-resolved manner from the institutional records, so we could apply neither absolute (systolic pressure < 100 mm Hg) nor relative (> 20% decline) criteria, and we could not examine the depth, duration or cumulative burden of hypotensive episodes. The initiation of a noradrenaline infusion reflects a clinical decision whose threshold may have varied between clinicians, between anaesthetic modalities and across the study period. Our exposure therefore identifies haemodynamic compromise judged clinically significant enough to treat, which is not interchangeable with the threshold-based definitions used in much of the perioperative and stroke literature; comparison with those studies should be made accordingly.

Third, the selection process constrains generalisability. Patients with unsuccessful recanalisation (TICI 0–1) were excluded by design, so our estimates apply only to patients in whom reperfusion was achieved and cannot be extrapolated to failed thrombectomy. Patients with incomplete peri-procedural haemodynamic records were also excluded; because record completeness may itself relate to clinical stability and to year of treatment, this exclusion may not have been random. As a single-centre series from a tertiary comprehensive stroke centre, the findings reflect one institution’s haemodynamic protocols and thresholds for vasopressor support and may not transfer to centres with different case volumes or practices.

Fourth, several procedural and anaesthetic variables were unavailable. Anaesthetic drug selection, depth of sedation, ventilatory parameters, procedure duration and the number of thrombectomy passes were not recorded systematically, and baseline ASPECTS was documented for only 35 patients (17.4%). The choice between general anaesthesia and conscious sedation was made clinically rather than at random, so the absence of an association with the exposure does not exclude a residual effect mediated by unmeasured aspects of anaesthetic conduct. Procedures were performed by five operators; although technique and device platform were institutionally standardised, operator identity was not recorded in the analytical dataset, and inter-operator variation in the threshold for further passes and in procedural conduct could not be quantified or adjusted for.

Fifth, the eight-year enrolment period (January 2017 to January 2025) saw substantial changes in thrombectomy technique, patient selection and blood-pressure management guidance, most notably following trials of intensive post-reperfusion blood-pressure lowering. Secular trends in practice may therefore have influenced both the exposure and the outcomes analysed.

Sixth, the association between active malignancy and the exposure rests on 18 affected patients, was not adjusted for tumour type, stage or oncological treatment, and did not reach the significance threshold within the subcohort with complete procedural time data. It should be regarded as hypothesis-generating.

Seventh, we assessed early in-hospital outcomes only. Functional status at 90 days measured by the mRS—the accepted primary endpoint in stroke intervention trials—and health-related quality of life were not evaluated.

Finally, the analysis of procedural delay was exploratory and undertaken after the primary results were known, in response to reviewers’ requests for a fuller description of the procedure; door-to-groin time was unavailable for 22 patients. No adjustment was made for multiple comparisons across the analyses reported here.

## 5. Conclusions

In summary, our study identifies post-procedural hypotension requiring vasopressor support as a common and clinically meaningful event after endovascular thrombectomy, occurring in more than one in four patients. It was associated with impaired early neurological recovery, with a shift in the entire day 7 modified Rankin Scale distribution towards greater disability, and with a more than two-fold higher in-hospital mortality. Its independent predictors were baseline stroke severity and active malignancy, whereas the anaesthetic technique showed no association with haemodynamic instability; in an exploratory analysis, a longer interval from arrival to arterial puncture was also independently associated with the exposure. These findings suggest that patient-level vulnerability, rather than the choice of anaesthesia, should guide peri-procedural haemodynamic strategy, and that patients presenting with severe stroke or active cancer may warrant intensified surveillance. Procedural delay was the only modifiable predictor identified and merits prospective evaluation. Prospective studies with standardised haemodynamic recording and 90-day functional follow-up are needed to confirm these associations and to establish whether preventing hypotension improves outcomes after EVT.

## Figures and Tables

**Table 1:** Baseline characteristics of the study population by post-procedural hypotension status (N = 201).

Variable	Total	Hypotension	No Hypotension	*p*
Age, years, mean ± SD	69.3 ± 14.3 (n = 201)	69.4 ± 12.7 (n = 55)	69.3 ± 14.9 (n = 146)	0.812
Age, median [IQR]	71 [61–79]	71 [64–76]	71 [61–80]	
Female sex	104/201 (51.7)	24/55 (43.6)	80/146 (54.8)	0.205
NIHSS on admission, mean ± SD	14.4 ± 5.1 (n = 199)	16.0 ± 5.0 (n = 55)	13.7 ± 5.0 (n = 144)	0.002
NIHSS on admission, median [IQR]	15 [11–18]	17 [13–20]	14 [11–17]	
General anaesthesia	140/201 (69.7)	40/55 (72.7)	100/146 (68.5)	0.609
Bridging intravenous thrombolysis	113/201 (56.2)	31/55 (56.4)	82/146 (56.2)	1.000
Successful reperfusion (TICI 2b–3)	198/201 (98.5)	54/55 (98.2)	144/146 (98.6)	1.000
Hypertension	152/200 (76.0)	44/55 (80.0)	108/145 (74.5)	0.463
Diabetes mellitus	56/200 (28.0)	17/55 (30.9)	39/145 (26.9)	0.599
Atrial fibrillation	84/200 (42.0)	23/55 (41.8)	61/145 (42.1)	1.000
Current smoking	28/200 (14.0)	9/55 (16.4)	19/145 (13.1)	0.648
Chronic kidney disease	22/200 (11.0)	9/55 (16.4)	13/145 (9.0)	0.203
Active malignancy	18/200 (9.0)	9/55 (16.4)	9/145 (6.2)	0.048

Footnote: One patient (no-hypotension group, conscious sedation) had no recorded comorbidity data; comorbidity denominators are therefore 200 overall and 145 in the no-hypotension group. NIHSS on admission was missing for 2 patients. Categorical comparisons by Fisher’s exact test; continuous by Mann–Whitney U.

**Table 2:** Neurological course and in-hospital outcomes by post-procedural hypotension status.

Outcome	Hypotension (n = 55)	No Hypotension (n = 146)	*p*-Value
NIHSS on admission	16.0 ± 5.0	13.7 ± 5.0	0.002 *
	17 (13–20)	14 (11–17)	
	[n = 55]	[n = 144]	
NIHSS, day 2	14.0 ± 6.9	9.4 ± 6.7	<0.001 *
	16 (8–20)	8 (4–13)	
	[n = 55]	[n = 137]	
NIHSS, day 7	12.3 ± 9.1	8.4 ± 9.0	0.003 *
	9 (5–19)	6 (2–12)	
	[n = 43]	[n = 125]	
mRS, day 7	3.8 ± 1.4	3.1 ± 1.7	0.023 *
	4 (3–5)	3 (2–4)	
	[n = 43]	[n = 125]	
Barthel Index, day 7	37.6 ± 37.6	49.0 ± 39.5	0.087
	20 (0–70)	50 (6–90)	
	[n = 43]	[n = 118]	
Functional independence (mRS 0–2), day 7, n (%)	7/43 (16.3)	37/125 (29.6)	0.108
In-hospital mortality, n (%)	28/55 (50.9)	30/146 (20.5)	<0.001 *

Note. Of 58 in-hospital deaths, 41 patients survived to day 7 and were assessed; 17 died before day 7. Continuous variables are presented as mean ± SD and median (IQR); the number of patients contributing to each comparison is given in brackets. mRS = modified Rankin Scale. * *p* < 0.05.

**Table 3:** Sensitivity analyses of day 7 outcomes.

Panel (A). Worst-Score Imputation (Death Before Assessment Assigned mRS 6, Barthel Index 0).				
Outcome	Hypotension	No Hypotension	*p*	*p* (Complete-Case)
Functional independence (mRS 0–2)	7/53 (13.2)	37/132 (28.0)	0.036 *	0.108
mRS at day 7	4.19 ± 1.57	3.27 ± 1.79	0.001 *	0.023
Barthel Index at day 7	30.5 ± 36.9	46.2 ± 40.0	0.010 *	0.087
**Panel (B). Ordinal Shift Analysis of Day 7 mRS, Death Coded as mRS 6 (n = 184).**				
**Variable**	**Common OR (95% CI)**		***p***	
Post-procedural hypotension	2.57 (1.40–4.72)		0.002 *	
Age (per year)	1.06 (1.03–1.08)		<0.001 *	
Female sex	1.24 (0.72–2.14)		0.435	
NIHSS on admission (per point)	1.11 (1.05–1.17)		<0.001 *	

Note. A common odds ratio above 1 indicates a shift in the entire modified Rankin Scale distribution towards greater disability. * *p* < 0.05.

**Table 4:** Univariable logistic regression—predictors of post-procedural hypotension.

Variable	OR (95% CI)	*p*-Value
Age (per 1 year)	1.00 (0.98–1.02)	0.963
Female sex	0.64 (0.34–1.19)	0.160
NIHSS on admission (per 1 point)	1.10 (1.03–1.17)	0.005 *
General anaesthesia (vs conscious sedation)	1.23 (0.62–2.44)	0.561
Hypertension	1.37 (0.64–2.93)	0.416
Diabetes mellitus	1.22 (0.62–2.40)	0.573
Atrial fibrillation	0.99 (0.53–1.86)	0.974
Current smoking	1.30 (0.55–3.07)	0.554
Chronic kidney disease	1.99 (0.80–4.95)	0.141
Active malignancy	2.96 (1.11–7.90)	0.031 *

Note. OR = odds ratio; CI = confidence interval. * *p* < 0.05.

**Table 5:** Patient and stroke characteristics by anaesthetic modality.

Variable	GA (n = 140)	CS (n = 61)	*p*-Value
Age, years	70.52 ± 14.37	66.57 ± 13.84	0.040 *
Female sex, n (%)	76 (54.3)	28 (45.9)	0.287
NIHSS on admission	14.74 ± 4.94	13.52 ± 5.31	0.163
Hypertension, n (%)	102 (72.9)	50 (83.3)	0.148
Atrial fibrillation, n (%)	65 (46.4)	19 (31.7)	0.061
Diabetes mellitus, n (%)	41 (29.3)	15 (25.0)	0.608
Current smoking, n (%)	17 (12.1)	11 (18.3)	0.270
Active malignancy, n (%)	15 (10.7)	3 (5.0)	0.282
Chronic kidney disease	13 (9.3)	9 (15.0)	0.323
Post-procedural hypotension, n (%)	40 (28.6)	15 (24.6)	0.609
In-hospital mortality, n (%)	50 (35.7)	8 (13.1)	0.001 *

Note. GA = general anaesthesia; CS = conscious sedation. * *p* < 0.05.

**Table 6:** Predictors of post-procedural hypotension.

Variable	Unadjusted OR (95% CI)	*p*	Adjusted OR (95% CI)	*p*
Age (per 1 year)	1.00 (0.98–1.02)	0.963	0.99 (0.97–1.02)	0.616
Female sex	0.64 (0.34–1.19)	0.160	0.57 (0.28–1.13)	0.106
NIHSS on admission (per point)	1.10 (1.03–1.17)	0.005	1.11 (1.04–1.19)	0.003 *
General anaesthesia (vs conscious sedation)	1.23 (0.62–2.44)	0.561	1.12 (0.54–2.34)	0.766
Chronic kidney disease	1.99 (0.80–4.95)	0.141	2.78 (1.00–7.70)	0.050
Active malignancy	2.96 (1.11–7.90)	0.031	2.90 (1.04–8.09)	0.042 *

Note. Multivariable model: n = 198, 55 events, 6 covariates (9.2 events per variable). Adjusted for all variables listed. OR = odds ratio; CI = confidence interval. * *p* < 0.05.

**Table 7:** Exploratory multivariable model including door-to-groin time (n = 177, 52 events).

Variable	Adjusted OR (95% CI)	*p*
Age (per year)	0.99 (0.96–1.01)	0.321
Female sex	0.60 (0.29–1.25)	0.173
NIHSS on admission (per point)	1.13 (1.05–1.22)	0.002
General anaesthesia (vs conscious sedation)	1.27 (0.56–2.87)	0.567
Chronic kidney disease	2.84 (0.95–8.50)	0.062
Active malignancy	2.78 (0.98–7.91)	0.056
Door-to-groin time (per 10 min)	1.10 (1.03–1.18)	0.003

Footnote: Exploratory analysis, not prespecified. Door-to-groin time was unavailable for 22 patients. For comparison, the same model fitted without door-to-groin time in these 177 patients gives: NIHSS 1.11 (1.03–1.19), *p* = 0.006; chronic kidney disease 2.36 (0.82–6.82), *p* = 0.111; active malignancy 2.69 (0.96–7.52), *p* = 0.059.

## Data Availability

The datasets generated and/or analysed during the current study are not publicly available due to privacy and ethical restrictions but are available from the corresponding author on reasonable request.

## Abbreviations

The following abbreviations are used in this manuscript:

aORs	Adjusted odds ratios
ASPECTS	Alberta Stroke Program Early CT Score
BP	Blood pressure
CS	Conscious sedation
EVT	Endovascular thrombectomy
GA	General anaesthesia
LVO	Large-vessel occlusion
mRS	Modified Rankin Scale
NIHSS	National Institutes of Health Stroke Scale
POQI	Perioperative Quality Initiative
SBP	Systolic blood pressure
TICI	Thrombolysis in Cerebral Infarction
CI	Confidence interval
CT	Computed tomography
IQR	Interquartile range
IRB	Institutional Review Board
MAP	Mean arterial pressure
OR	Odds ratio
SD	Standard deviation
SPSS	Statistical Package for the Social Sciences
